# Supplementary material for: Cold adaptation in the environmental bacterium Shewanella oneidensis is controlled by a J-domain co-chaperone protein network
Source: Commun Biol. 2019 Aug 29;2:323. doi: 10.1038/s42003-019-0567-3 (PMC6715715; doi:10.1038/s42003-019-0567-3)
Supplement: Supplementary file 1 — Supplementary Information [file 42003_2019_567_MOESM1_ESM.pdf]

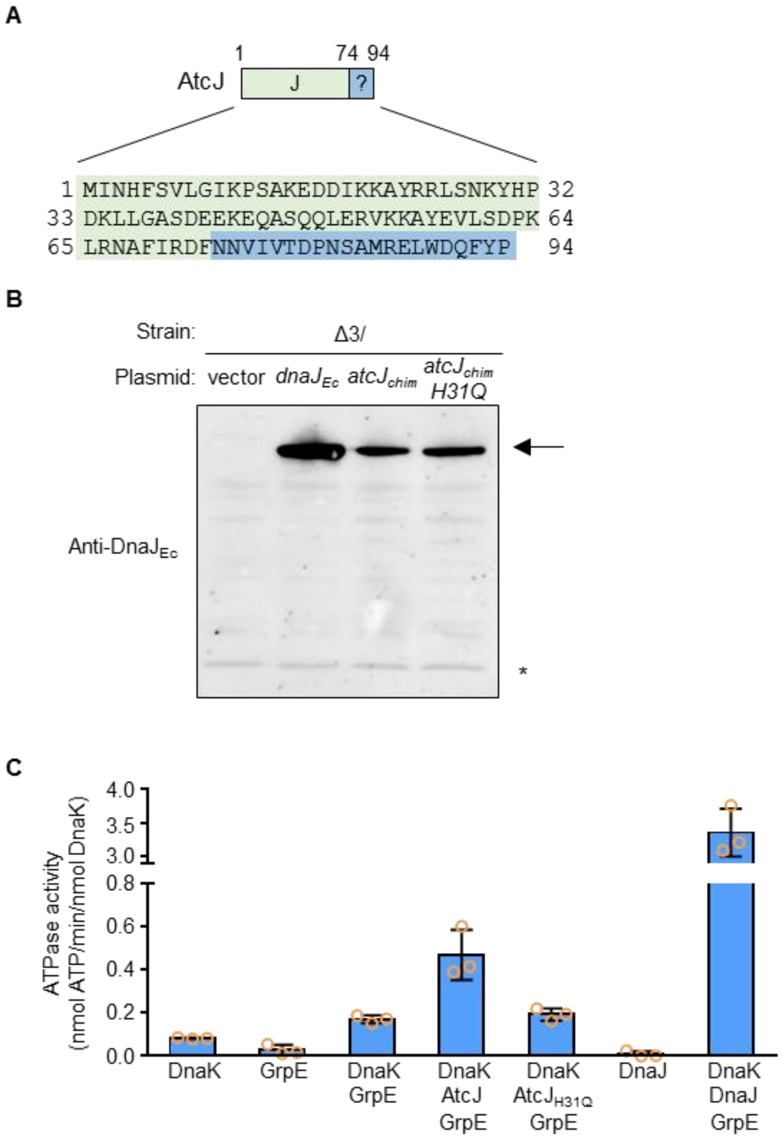

Supplementary Figure 1: AtcJ sequence, chimera production, and ATPase assays (A) Protein sequence of AtcJ. The J-domain is shown in light green and the C-terminal extension of 21 amino acids in blue. **(B)** Western-blot to check that the wild-type and mutant chimera were produced in similar amount. *E. coli* Δ3 strain containing the pBad33 vector, or plasmids producing $DnaJ_{Ec}$, $AtcJ_{chim}$, or $AtcJ_{chimH31Q}$ were grown at 28°C in LB media supplemented with chloramphenicol and 0.2% L-arabinose until stationary phase. Protein extracts were analyzed by Western blot using an anti-$DnaJ_{Ec}$ antibody. The arrow indicates the bands corresponding to $DnaJ_{Ec}$, $AtcJ_{chim}$ or $AtcJ_{chimH31Q}$ mutant. The star (*) indicates a contaminant protein used here to show that the same amount of the different extracts was loaded on the gel. **(C)** Stimulation of the DnaK ATPase activity by AtcJ in the presence of GrpE. ATPase activities were measured at 37°C using an enzyme-coupled spectrophotometric ATPase assay with 10 µM DnaK, 2 µM GrpE, 50 µM AtcJ or $AtcJ_{H31Q}$, or 2 µM DnaJ where indicated. Data from three replicates are shown as mean ± SD.

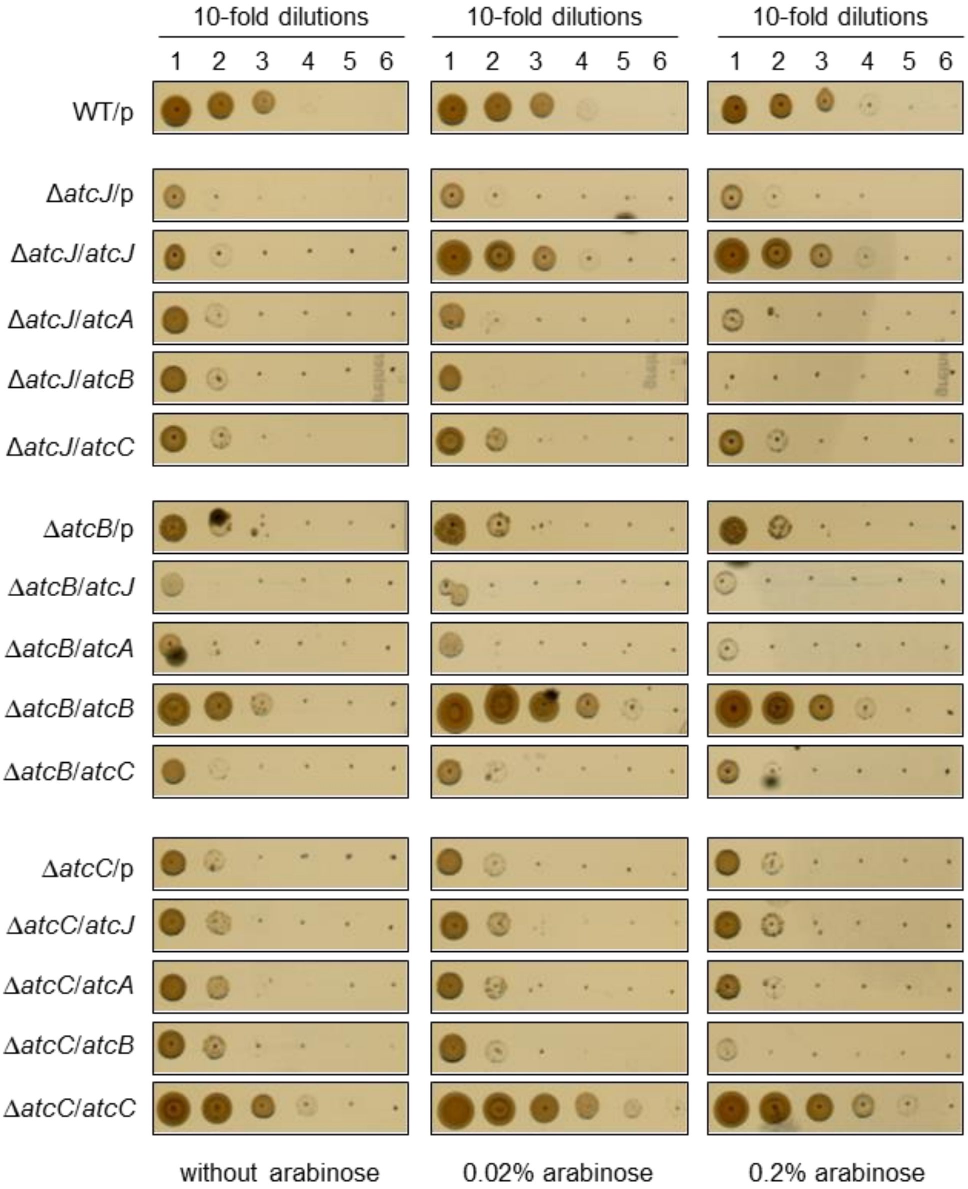

Supplementary Figure 2: Complementation of the growth phenotypes at low temperature is specific. After initial growth at 28°C, wild-type, *ΔatcJ*, *ΔatcB*, or *ΔatcC* strains containing as indicated the pBad33 vector (p) or the plasmids producing AtcJ, AtcA, AtcB, or AtcC were diluted to OD_{600}=1. 10-time serial dilutions were spotted on LB-agar plates containing 0%, 0.02% or 0.2% L-arabinose. Plates were incubated 10 days at 7°C. The experiment shown is representative of 3 independent experiments.

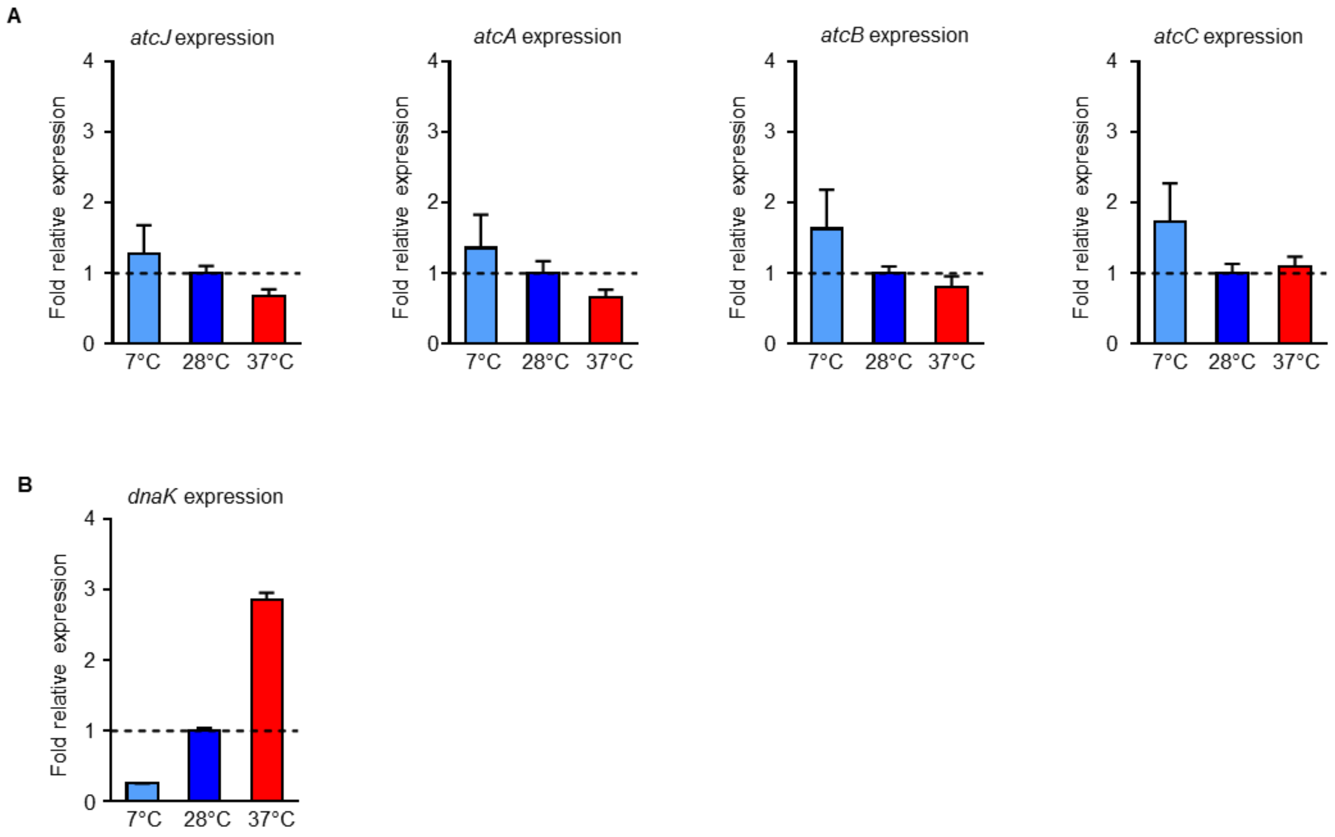

Supplementary Figure 3: *atcJ* expression is not induced at low temperature. (A-B) Wild-type *S. oneidensis* strain was grown at 7°C, 28°C, or 37°C until OD_{600}=2, total RNA was extracted, retro-transcribed in cDNA, and quantitative-PCR was performed using specific oligonucleotides to amplify *atcJ*, *atcA*, *atcB*, and *atcC* (A) or *dnaK* (B). Data are shown as fold variation of expression relative to 28°C. Dot line (y=1) indicates the relative expression value for which there is no variation compared to the expression at 28°C. Data from at least two replicates are shown as mean ± SEM.

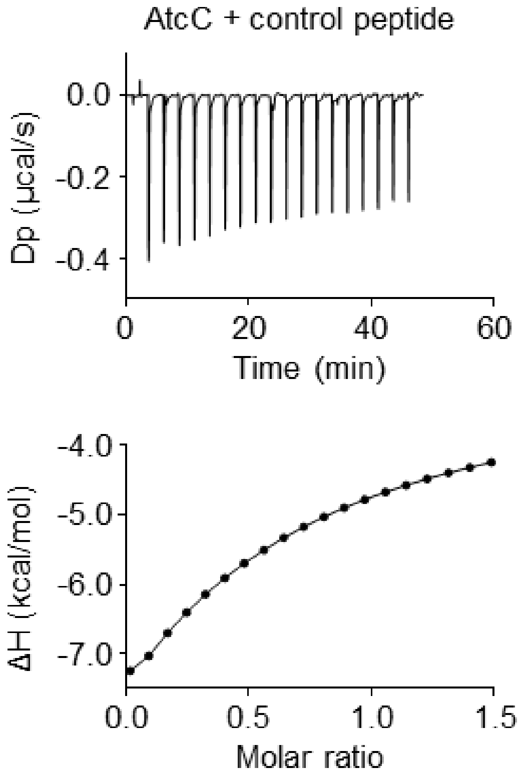

Supplementary Figure 4: A random peptide does not interact with AtcC. ITC experiments were performed at 25°C with 36 µM AtcC and 285 µM of the $pep_{control}$ peptide with the SRSSLASAWGRFLLQRGSWTGPRC sequence. Top panels show heat exchange upon ligand titration and bottom panels show integrated data with binding isotherms fitted to a single-site binding model. Data shown are representative of two independent experiments.

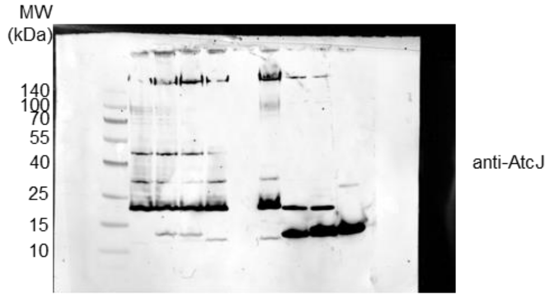

Full-length blot from figure 2D

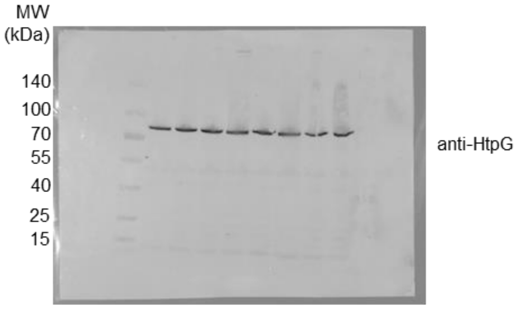

Full-length blot from figure 2D

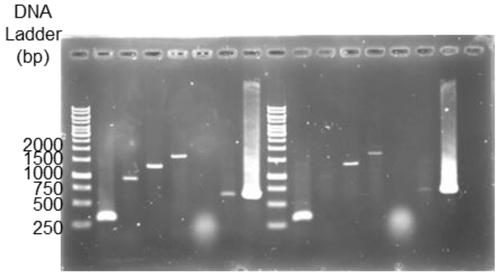

Full-length agarose gel from figure 3B

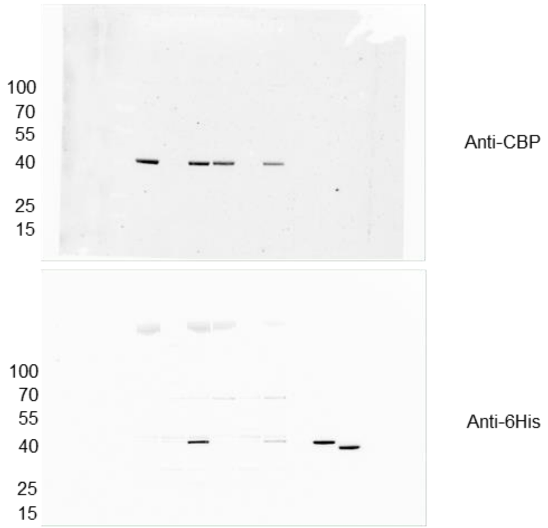

Full-length blots from figure 5B

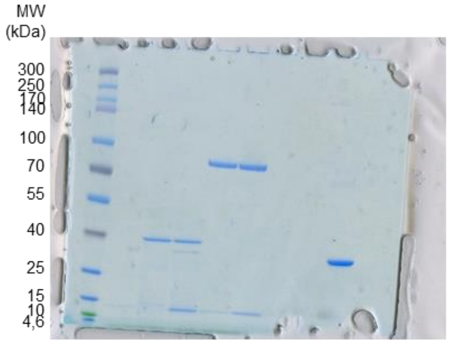

Full-length gel from figure 5C

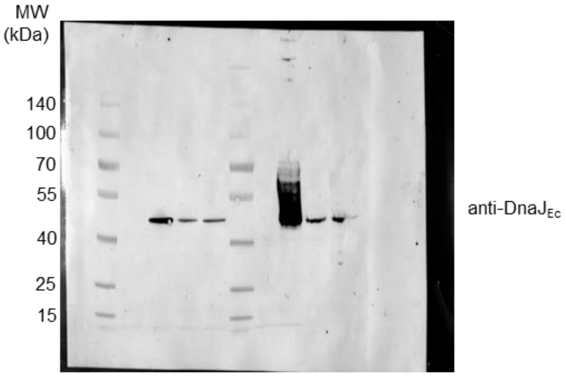

Full-length blot from figure S1B

Supplementary Figure 5: Full-length blots and gels showed in the figures of the paper. The related figures of the paper are indicated.

Supplementary Table 1: DNA primers used in this study.

Restriction site	Primer sequence	Usage	
BamHI	GAAGGATCCAGAGTTGCAATATGGCTGCTCGTCGG	To amplify a 500 pb fragment upstream of *atcJ*	To delete *atcJ*
	TCAAAATAAATAATTTGTACCTGCAGGCGATGGGTTGG		
	GCAGGTACAAATTATTTATTTTGAGTACCAAATAAACG	To amplify a 500 pb fragment downstream of *atcJ*	
SpeI	GAAACTAGTTAAGTAAAGCACGGCGCAGCTTTC		
BamHI	TTAGGATCCGATCCATCGCTGACTAATCATAATTC	To amplify a 500 pb fragment upstream of *atcA*	To delete *atcA*
	CCTCATCATCGTGTCTCGAAATATAAGCGCTAAAAC		
	ATATTTCGAGACACGATGATGAGGACCGCTAATGAAC	To amplify a 500 pb fragment downstream of *atcA*	
SpeI	TAACTAGTGGTGTCGGTATCTTCTTCATCGTCG		
ApaI	TTAGGGCCCGAGCTTAAAACCAATGCCTACGAC	To amplify a 500 pb fragment upstream of *atcB*	To delete *atcB*
	TTAATCTAAAAATTAGCGGTCCTCATCATCGTAGTG		
	GAGGACCGCTAATTTTTAGATTAAGGATCCCTAGATG	To amplify a 500 pb fragment downstream of *atcB*	
SpeI	TAACTAGTTCAGCGCCGCAATCGATTGCTGGCG		
ApaI	TTAGGGCCCAATCAAAAACAATGCCGAAGAACTC	To amplify a 500 pb fragment upstream of *atcC*	To delete *atcC*
	CCCTGATGAGGACTAGGGATCCTTAATCTAAAAAGC		
	TTAAGGATCCCTAGTCCTCATCAGGGGCGTTAGTCC	To amplify a 500 pb fragment downstream of *atcC*	
SpeI	TAACTAGTAGCTTGTCGCAATGAATGCAGACAC		
NdeI	TACATATGGGTAAAATTATTGGTATCGACTTAGG	To construct pET-$DnaK_{So}$	
SacI	TAGAGCTCTTAGTGGTGGTGGTGGTGGTGTTTCTTGTCGTCTTTCACTTCTTC		
NdeI	TACATATGTCAAAGCGAGATTATTACGAAGTATTAG	To construct pET-$DnaJ_{So}$	
SacI	TAGAGCTCTTAGTGGTGGTGGTGGTGGTGGCTATTTAGGTCTTGGAAGAACTTC		
NdeI	TACATATGCACCACCACCACCACCACATGAGCAACGAGTCGATTAAAGC	To construct pET-$GrpE_{So}$	
SacI	TAGAGCTCTTAGGCTTCAATATCAATCGACGC		
NdeI	TACATATGATTAACCACTTTAGTGTGCTTGG	To construct pET-AtcJ	
SacI	TAGAGCTCTTAGTGGTGGTGGTGGTGGTGAGGGTAGAACTGATCCCATAATTC		
NdeI	TACATATGCAACTGGTACTGCGCGATATAG	To construct pET-AtcC	
SacI	TAGAGCTCTTAGTGGTGGTGGTGGTGGTGCAGCACGATATAAAGCGGCCAGC		
NdeI	TACATATGAACACGAGTCTGATTGAGATAAC	To construct pET-AtcB	
EcoRI	TAGAATTCTTAGTGGTGGTGGTGGTGGTGATCTAAAAAGCTAAACATATC		
KpnI	TAGGTACCAGATGAACACGAGTCTGATTGAGATAAC	To construct pET52-AtcB	
SalI	TAGTCGACTTAATCTAAAAAGCTAAACATATC		
KpnI	TAGGTACCAGATGTCACAACAAGAAACTCATGG	To construct pET52-$HtpG_{So}$	
SacI	TAGAGCTCTTACTTCAGGTTCGCCAATAAC		
EcoRI	TAGAATTCATGATTAACCACTTTAGTGTG	To construct pT18-AtcJ	
XhoI	TACTCGAGTTAAGGGTAGAACTGATCCCATAATTCGC		
EcoRI	TAGAATTCATGAACACGAGTCTGATTGAG	To construct pT18-AtcB and pT25-AtcB	
XhoI	TACTCGAGTTAATCTAAAAAGCTAAACATATC		
EcoRI	TAGAATTCATGCAACTGGTACTGCGCGATATAG	To construct pT18-AtcC and pT25-AtcC	
XhoI	TACTCGAGTTACAGCACGATATAAAGCGGCCAGCC		
PstI	TACTGCAGAAATGCCCATGGCCAAAAAACTG		

XbaI	TATCTAGATTAGCGGTCCTCATCATCGTAG	To construct pT25-AtcA	
XmaI	TACCCGGGGAAGGAGATATACATATGATTAACCACTTTAGTGTGCTTGG	To construct pBad33-AtcJ	
XbaI	TATCTAGATTAAGGGTAGAACTGATCCCATAATTCGC		
XmaI	TACCCGGGGAAGGAGATATACATATGGCTAAGCAAGATTATTACGAG	To construct pBad33-DnaJ$_{Ec}$	
XbaI	TATCTAGATTAGCGGGTCAGGTCGTCAAAAAAC		
XmaI	TACCCGGGGAAGGAGATATACATATGATTAACCACTTTAGTGTGCTTGG	To construct AtcJ chimera	
	CAAACGCAGCATGGAAATCTCGGATAAACGCATTTC		
	ATCCGAGATTTCCATGCTGCGTTTGAGCAAGGTGGC		
XbaI	TATCTAGATTAGCTATTTAGGTCTTGGAAGAAC		
	TCAAACAAATATCAACCTGACAAGTTG	To obtain AtcJ$_{H31Q}$	
	CAACTTGTCAGGTTGATATTTGTTTGA		
	ATCCGAGATTTCTAGAACGTGATAGTG	To obtain AtcJ$_{\Delta C}$	
	CACTATCACGTTCTAGAAATCTCGGAT		
	CGCCGGCTCTCAAACAAATA	qRT-PCR of *atcJ*	
	TTGGGGTCGGTCACTATCAC		
	GCCAAGCTGTAACAAACCCA	qRT-PCR of *dnaK*	
	CGTGACTCAACCCAAGCATC		
	GGCTGACCCTAAAATGCTCG	qRT-PCR of *atcA*	
	CGTACCGCCAACACTAAAGG		
	ACAGGCTAGCAAACGTTTCG	qRT-PCR of *atcB*	
	TGCTCAGGAGTCATCAGCTC		
	GCGTGGTTAAGTGCTTCCAA	qRT-PCR of *atcC*	
	GCGTTGGTGCTCGACTAAAG		
