# Supplementary material for: Cold adaptation in the environmental bacterium Shewanella oneidensis is controlled by a J-domain co-chaperone protein network
Source: Commun Biol. 2019 Aug 29;2:323. doi: 10.1038/s42003-019-0567-3 (PMC6715715; doi:10.1038/s42003-019-0567-3)
Supplement: Supplementary file 2 — Description of Additional Supplementary Files [file 42003_2019_567_MOESM2_ESM.docx]

**Description of additional supplementary items**

**Supplementary Data 1:** Source data underlying the graphs and charts presented in the main figures.
