# Supplementary material for: Cold adaptation in the environmental bacterium Shewanella oneidensis is controlled by a J-domain co-chaperone protein network
Source: Commun Biol. 2019 Aug 29;2:323. doi: 10.1038/s42003-019-0567-3 (PMC6715715; doi:10.1038/s42003-019-0567-3)
Supplement: Supplementary file 4 — Reporting Summary [file 42003_2019_567_MOESM4_ESM.pdf]

nature research

Corresponding author(s): Olivier Genest

Last updated by author(s): Jul 24, 2019

Reporting Summary

Nature Research wishes to improve the reproducibility of the work that we publish. This form provides structure for consistency and transparency in reporting. For further information on Nature Research policies, see Authors & Referees and the Editorial Policy Checklist.

Statistics

For all statistical analyses, confirm that the following items are present in the figure legend, table legend, main text, or Methods section.

n/a	Confirmed	
☐	☒	The exact sample size (n) for each experimental group/condition, given as a discrete number and unit of measurement
☐	☒	A statement on whether measurements were taken from distinct samples or whether the same sample was measured repeatedly
☒	☐	The statistical test(s) used AND whether they are one- or two-sided<br>*Only common tests should be described solely by name; describe more complex techniques in the Methods section.*
☒	☐	A description of all covariates tested
☒	☐	A description of any assumptions or corrections, such as tests of normality and adjustment for multiple comparisons
☐	☒	A full description of the statistical parameters including central tendency (e.g. means) or other basic estimates (e.g. regression coefficient) AND variation (e.g. standard deviation) or associated estimates of uncertainty (e.g. confidence intervals)
☒	☐	For null hypothesis testing, the test statistic (e.g. F, t, r) with confidence intervals, effect sizes, degrees of freedom and P value noted<br>*Give P values as exact values whenever suitable.*
☒	☐	For Bayesian analysis, information on the choice of priors and Markov chain Monte Carlo settings
☒	☐	For hierarchical and complex designs, identification of the appropriate level for tests and full reporting of outcomes
☒	☐	Estimates of effect sizes (e.g. Cohen's d, Pearson's r), indicating how they were calculated

Our web collection on statistics for biologists contains articles on many of the points above.

Software and code

Policy information about availability of computer code

Data collection	Bio-Rad CFX Manager software, version 3.1<br>PEAQ-ITC Analysis Software, Malvern<br>Tecan SPARKCONTROL
Data analysis	Microsoft Excel, 2016<br>Graph Pad Prism 7.03<br>Photoscape 3.7<br>Adobe Photoshop CS6<br>EPSON SCAN ver 3.9.2.1 FR<br>Bio-Rad CFX Manager software, version 3.1<br>PEAQ-ITC Analysis Software, Malvern<br>Expasy BoxShade : https://embnet.vital-it.ch/software/BOX_form.html<br>Pymol

For manuscripts utilizing custom algorithms or software that are central to the research but not yet described in published literature, software must be made available to editors/reviewers. We strongly encourage code deposition in a community repository (e.g. GitHub). See the Nature Research guidelines for submitting code & software for further information.

Data

Policy information about availability of data

All manuscripts must include a data availability statement. This statement should provide the following information, where applicable:

- Accession codes, unique identifiers, or web links for publicly available datasets
- A list of figures that have associated raw data
- A description of any restrictions on data availability

The authors declare that the data supporting the findings of this study are available within the paper and its supplementary information files.

Field-specific reporting

Please select the one below that is the best fit for your research. If you are not sure, read the appropriate sections before making your selection.

☒ Life sciences ☐ Behavioural & social sciences ☐ Ecological, evolutionary & environmental sciences

For a reference copy of the document with all sections, see nature.com/documents/nr-reporting-summary-flat.pdf

Life sciences study design

All studies must disclose on these points even when the disclosure is negative.

Sample size	N/A
Data exclusions	No data was excluded
Replication	All experiments were done at least in triplicate and when applicable results were given as mean +/- standard deviation
Randomization	N/A
Blinding	N/A

Reporting for specific materials, systems and methods

We require information from authors about some types of materials, experimental systems and methods used in many studies. Here, indicate whether each material, system or method listed is relevant to your study. If you are not sure if a list item applies to your research, read the appropriate section before selecting a response.

Materials & experimental systems

n/a	Involved in the study	
☐	☒	Antibodies
☒	☐	Eukaryotic cell lines
☒	☐	Palaeontology
☐	☒	Animals and other organisms
☒	☐	Human research participants
☒	☐	Clinical data

Methods

n/a	Involved in the study	
☒	☐	ChIP-seq
☒	☐	Flow cytometry
☒	☐	MRI-based neuroimaging

Antibodies

Antibodies used

Anti-AtcJ: produced in rabbit from purified full length AtcJ protein from Shewanella oneidensis (Biotem http://www.biotem.fr/ France). Validated by western blots on purified proteins.

Anti-CBP: Anti-Calmodulin Binding Protein Epitope Tag Antibody, MERCK, # 07-482

Anti-HIS: 6x-His Tag Monoclonal Antibody (AD1.1.10), HRP. ThermoFisher # MA1-80218

Anti-HtpG: produced in rabbit from purified full length HtpG protein from Escherichia coli. Validated by western blots on purified proteins.

Anti-DnaJ: produced in rabbit from purified full length DnaJ protein from Escherichia coli. Validated by western blots on purified proteins.

Validation

Anti-AtcJ: Validated by Western blots with purified proteins.

Anti-CBP: Validated by MERCK for western blot applications.
Anti-6HIS: Validated by INVITROGEN for western blot applications.
Anti-HtpG: Validated by Western blots with purified proteins.
Anti-DnaJ: Validated by Western blots with purified proteins.

Animals and other organisms

Policy information about studies involving animals; ARRIVE guidelines recommended for reporting animal research

Laboratory animals	Bacteria: Shewanella oneidensis MR1 wild type and mutants as described; Escherichia coli K12 wild type and mutants as described.
Wild animals	N/A
Field-collected samples	N/A
Ethics oversight	N/A

Note that full information on the approval of the study protocol must also be provided in the manuscript.
